# Somatic screening and lifestyle factors in adolescents with psychiatric disorders: results of a field-based prevalence study

**DOI:** 10.1192/j.eurpsy.2026.11147

**Published:** 2026-07-31

**Authors:** E. Klopper

**Affiliations:** Child and adolescent psychiatry, Community mental health centre GGNet, Doetinchem, Netherlands

## Abstract

**Introduction:**

Somatic disorders are highly prevalent among psychiatric patients, increasing the risk of cardiovascular and metabolic complications and contributing to reduced life expectancy. Although many psychiatric disorders emerge during adolescence (ages 12–24), research on somatic comorbidities and lifestyle factors in this group remains limited. The lack of validated somatic screening tools for adolescents with psychiatric conditions further hinders early detection and preventive care.

**Objectives:**

To evaluate the effectiveness of standardized somatic screening in adolescent psychiatric outpatients and to identify the prevalence of somatic and lifestyle-related risk factors using the newly developed *Somatic Mini Screen Youth (SmS-Y).*

**Methods:**

This field-based cross-sectional study included 153 adolescents (mean age = 17.8 years; 62.7% female) with various psychiatric disorders. The SmS-Y comprised a structured questionnaire, somatic measurements, and routine blood tests. Real world data on psychiatric diagnoses, somatic comorbidities, medication use, lifestyle habits, and laboratory findings were analyzed descriptively.

**Results:**

Mood disorders were the most common diagnosis (61.4%), followed by ADHD (43.8%), anxiety and trauma-related disorders (43.1%), and autism spectrum disorders (39.2%). A minority (35.9%) used psychopharmaceuticals, while 26.8% took somatic medication or vitamin/mineral supplements. Somatic comorbidities occurred in 46.9% of cases, most frequently lung problems (13.7%) such as asthma, and skin problems (11%) such as eczema. Additionally, 32.7% had known allergies. Caffeine use was reported in 57.6%, alcohol in 32.9%, smoking or vaping in 19.6%, and substance use in 15% (mainly cannabis, 12.4%). Regarding somatic parameters, 58.6% had normal weight, 15% were overweight, and 9.8% obese. Normal waist circumference, blood pressure, and resting heart rate were found in 68.7%, 73.5%, and 61.2%, respectively. Blood tests revealed lipid abnormalities in 28.4%, deviated prolactin in 24.5%, vitamin D deficiency in 43.9%, thyroid abnormalities in 2%, and vitamin B12 abnormalities in 7.8%.

Overall, 81.7% of screenings identified health concerns requiring treatment adjustments, including lifestyle interventions (81%) and medical management (74%), such as medication adjustments, nutritional supplements, or referrals to general practitioners or specialists.

**Conclusions:**

These findings underline the importance of routine somatic screening in adolescent psychiatric care. Integrating such screenings enables early detection of physical health issues, informs treatment, and supports preventive strategies. Simple lifestyle interventions can yield major health benefits and add healthy life years to this vulnerable population—the next generation.

**Disclosure of Interest:**

None Declared

